# Butterflies in the stomach: a critical analysis on human scoleciasis

**DOI:** 10.1093/trstmh/traf095

**Published:** 2025-09-19

**Authors:** Michele Calatri

**Affiliations:** Faculty of Medicine and Surgery, University of Cagliari, Cagliari, Italy

**Keywords:** butterfly, caterpillar, moth, parasitic infestation, pseudoparasitosis, scoleciasis

## Abstract

Butterflies and moths have been admired for their beauty since ancient times, but even these graceful insects can pose a danger to humans, albeit rarely, mainly because of the stinging toxic hairs on the larval stage of some species. In addition to this, since the 16th century, occasional findings of caterpillars still alive after being expelled by people through vomit or faeces led prominent scientists to consider the possibility that the larvae of some species of butterflies and moths, if accidentally ingested, could survive in the human gastrointestinal tract and cause a true infestation. More recently, in the 20th century, there have been reports of caterpillars of certain moths penetrating pre-existing skin wounds under particular circumstances. The human infestation (true or alleged) with caterpillars is known as scoleciasis. The objective of this study is to provide a comprehensive review of all documented cases of human scoleciasis in the literature to date and to assess whether or not this phenomenon should be considered a true parasitosis.

## Introduction

Butterflies and moths belong to the Lepidoptera, an order of winged insects that includes >165 000 species worldwide. The development of these holometabolous insects has four stages: it begins with the egg, which gives rise to a wormlike polypod larval form known as a caterpillar, whose only activity is to feed until, after four or five moults, it reaches its maximum development and then pupates in a silky cocoon before emerging as an adult or ‘imago’. The caterpillars of most species are phytophagous, while some feed on other animal products, but regardless of their diet, many are considered crop and other primary food pests due to their voracious appetites. Adult lepidopterans feed mainly on nectar but also on other sugary substances that they can find in a wide variety of substrates and a few species feed on tears, blood and sweat of various animals, including humans, to supplement dietary sodium intake. Although these particular feeding behaviours are certainly interesting from a biological point of view, it has no medical importance to date.^1^ Very rare cases have been reported where adult moths have somehow managed to enter cavities in the human body through natural orifices, but again these findings have had no significant effect on humans other than a slight annoyance.^2^ A little more than 100 species of caterpillars and some adults have developed special defence mechanisms to protect themselves from predators, such as toxic stinging hairs, poisonous spines and repulsive secretions.^3^ In humans, exposure to the substances produced by these species can cause a variety of adverse reactions, ranging from simple skin irritation to fatal toxic shock.^4,5^

In the scientific literature, two terms are commonly used to describe medical issues caused by Lepidoptera in humans:

- ‘lepidopterism’, from the Greek ‘λεπις’ (lepis) = scales, ‘πτερόν’ (pteron) = wing and ‘ισμός’ (ism) = condition, is a word that generically identifies all adverse reactions caused in humans by butterflies and moths and their caterpillars and cocoons but is often used specifically in those cases where adults capable of secreting toxic substances are involved;^6^ and
- ‘erucism’, from ‘ἔρυξ’ (eryx) = caterpillar and ‘ισμός’ (ism) = condition, which is instead the correct term when talking about adverse reactions caused specifically by the urticating hairs of some caterpillars and cocoons.^7^

A third negative interaction between lepidopterans and humans is expressed in the ability of some caterpillars to invade the human body and cause a real or presumptive infestation, analogous to what the larvae of some dipterans can do in myiasis. This little-known condition is commonly known in the scientific literature as ‘scoleciasis’ or also ‘scolechiasis’ or ‘scholechiasis’, from ‘σκώληξ’ (scolex) = worm and ‘ᾱσις’ (asis) = diseases.

The term first appeared in 1815, in the William Kirby and William Spence work ‘Introduction to Entomology’ to define all those infestations in humans caused generically by larvae of insects.^8^ It was another English entomologist, Frederick William Hope, who 25 y later disambiguated the original term: he assigned the term ‘canthariasis’ to human infestations involving beetle larvae, ‘myiasis’ to those caused by dipteran larvae and ‘scolechiasis’ in those involving caterpillars.^9^

## History

**Figure 1.**
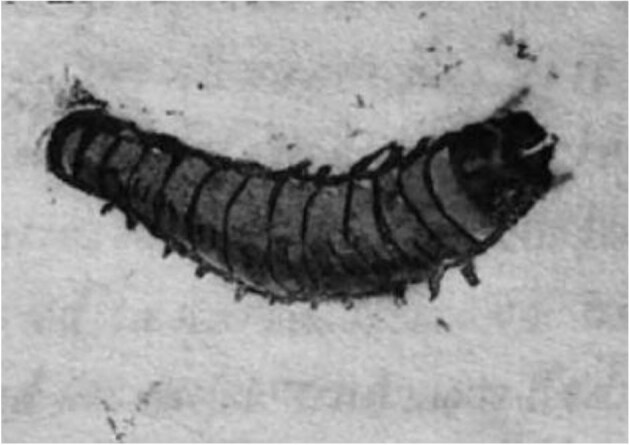
The front cover of Angelini's 1610 publication featured a drawing of a caterpillar expelled by a boy.

The earliest known descriptions of presumed human infestations by caterpillars date back to the 16th century in Europe. Although still not detailed, these accounts were written by prominent scientists of the time such as Ambroise Paré, the physician and surgeon to King Henry II of France and one of the founders of modern surgery. In his volume ‘*Des monstres et prodiges*’, Paré recounts the case of a 40-year-old woman who, in October 1578, vomited up ‘…three hairy worms similar in shape, colour, length and size to caterpillars’.^10^ A few years later, in 1581, Rembert Dodoens, a Flemish physician, in the work ‘*Medicinalium observationum exempla rara*’, recalled the case of ‘…a nine-year-old girl, who had been given a vermifuge medicine, which expelled worms not resembling earthworms, but resembling caterpillars, being they shorter, with more feet and still alive’.^11^ However, the first reliable description of a possible human infestation sustained by a lepidopteran larva was given in a brief Latin account published in 1610 in Ravenna by the Italian physician and philosopher Fulvio Angelini. It concerns a 34-year-old man who complained of fever, headache and nasal congestion. In front of the doctor, he expelled with a blood clot ‘a worm, as long as half a finger, with a large body, red, with a black head, and hard…, endowed with some red hairs, six feet towards the head, but having some small ones towards the tail, by which it walked quietly’.^12^ The ‘worm’ remained alive for 4 d and the author drew a picture of it on the front cover of his publication (Figure 1).

The first English-language publication on the subject was written by Martin Lister, a physician and naturalist. In a letter addressed to the Royal Society of London, published in July 1675, Lister describes a case of a 9-year-old boy from Yorkshire who had suffered from severe stomach cramps for several days before vomiting worms, which were then examined by Lister: ‘…These worms were very caterpillars with fourteen legs, six small pointed, the eight middle stumps, and the two hind claspers; something more than an inch long, and of the thickness of a ducks-quill, thin haired or rather naked, with brown annuli and a black head.…The very same for kind that I have many times seen on plants, and no doubt, these would in due time have shrunk into chrysalis, and changed into moths’.^13^

**Figure 2.**
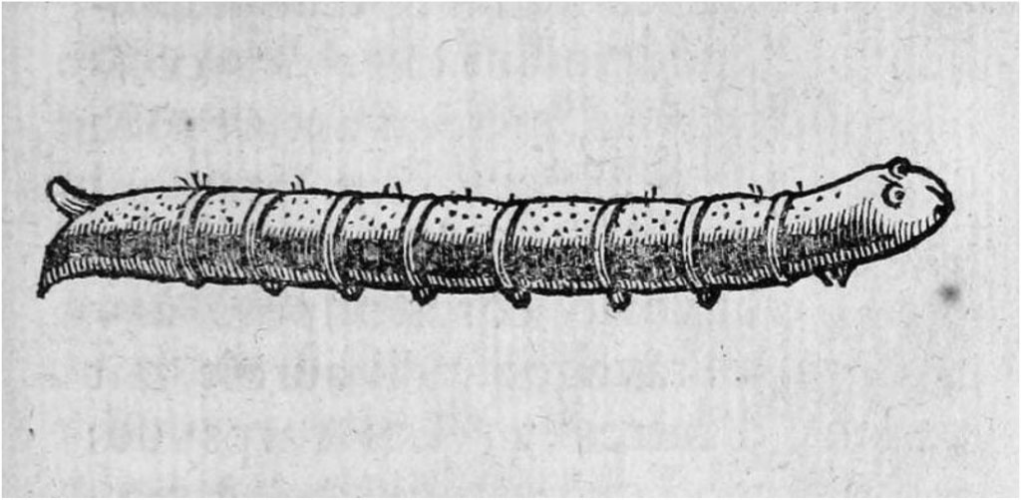
Drawing of the caterpillar expelled by the Vicar General of the Diocese of Alais in 1741.

A century after Angelini's drawing, the Vicar General of the Diocese of Alais, in France, provided another illustrated account. This was reported in 1741 in the book ‘*De la generation des vers dans le corps de l'homme*’ by Nicolas Andry De Bois-Regard, a French physician and man of letters who is considered by many to be one of the fathers of modern parasitology. The priest described the insect he had vomited (Figure 2) as ‘black, with caterpillar legs, a little hairy’, and regarding the species, assumed it was those ‘‘worms’…which are present in textiles and food’.^14^

Another interesting case, with some witnesses, was the one that the physician Vetillard described to the famous French naturalist Georges-Louis Leclerc, Comte de Buffon. This was a particular episode dating back to 1761 involving a tubercular woman in her 30s from Mans who, following a ‘crisis’, had expelled with her vomit a living insect that was taken to the woman's personal physician, Vetillard, who, given the exceptional nature of its discovery, decided to keep it with him. It was, he wrote, ‘a caterpillar…brown with blacker longitudinal stripes, had sixteen legs and walked like other caterpillars; it had small tufts of hair, especially on the body rings. The head was black, shiny and scaly, divided by a groove into two equal parts, which could be mistaken for two eyes’.^15^

**Figure 3.**
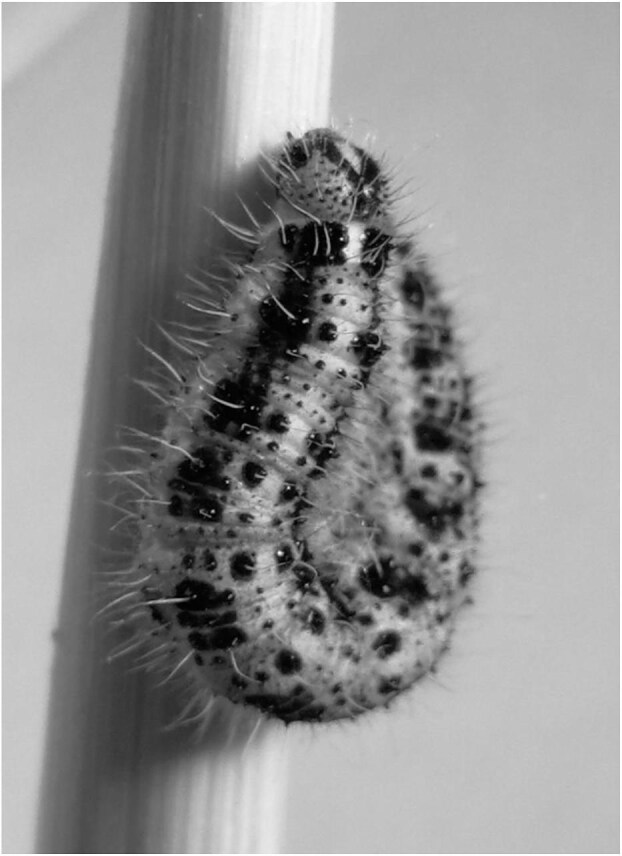
*P. brassicae* caterpillar.

A particularly impressive case was described by Scottish surgeon Robert Calderwood in a letter to his colleague Andrew Duncan, published in the ninth volume of ‘*Medical and Philosophical Commentaries*’ in 1783.^16^ Calderwood reported on a 4-year-old boy who experienced general malaise, headaches, nocturnal teeth grinding and abdominal pain, which eventually progressed to a comatose state. After being treated with a purgative, the child passed some insect larvae in his faeces. These were then sent to Duncan for examination, who described them as ‘much resembling the common caterpillar’, probably the reference is to the caterpillar of the ‘large white’ or cabbage butterfly, *Pieris brassicae* L. (Lepidoptera: Pieridae) (Figure 3). The parents reported that these larvae continued to actively creep out from their son's anus throughout the night, scattering in various places around the room. Calderwood was sceptical about this, at least until he saw the phenomenon with his own eyes, when he visited the child again the next day: ‘Having never seen or heard of such animals in the human body, I would hardly believe he had passed them. To convince me, they turned down the bed clothes, when, to my great astonishment, they were to be seen in numbers about his breech, three or four having made their way up to the pillow’.^16^

In 1830, André Marie Constant Dumeril, a well-known French zoologist, reported the case of a woman who expelled two caterpillars of the genus *Noctua* L. (Lepidoptera: Noctuidae) through the anus after taking a purgative to combat constipation.^17^

About 60 y later, in 1896, M. Poujade reported a case involving the grease moth *Aglossa pinguinalis* Linnaeus (Lepidoptera: Pyralidae): a father and his 7-year-old son from the Haute-Marne region of France had expelled caterpillars through vomiting. In the child, the caterpillars ‘had entered the larynx and clung to it, making their removal very difficult’.^18^

The first case in which caterpillars were not associated with gastrointestinal symptoms dates to the early 20th century. In August 1936, an article titled ‘A case of infestation with the corn borer, *Pyrausta nubilalis* (scoleciasis)’ was published in the Canadian Medical Association Journal by B. Church.^19^ He described a case he had personally followed, involving a 58-year-old woman who complained of intense vaginal itching and irritation. These symptoms had resulted in an inability to urinate and increasing irritability. Church then performed a physical examination that revealed ‘…three symmetrical oedematous blebs around the orifice of the urethra, with what appeared to be a whitish mucous plug in the opening itself. On catching this plug and pulling on it, a live worm about three-quarters of an inch long was extracted. Once the plug was removed a large quantity of urine followed, which had evidently been under some pressure. There was immediate relief and the itching and discomfort soon began to disappear’. A few days later the Entomological Branch of the Department of Agriculture in Ottawa identified the caterpillar as belonging to the European corn borer, *Pyrausta* (now *Ostrinia*) *nubilalis* Hubner (Lepidoptera: Crambidae).

In 1962, Harold George Scott, an official of the United States Public Health Service and a biologist, compiled a list of 120 cases of insect larvae infestations in humans reported in North America between 1952 and 1962. Among these cases, four were scoleciasis^20^: the first one describes briefly a case involving a noctuid caterpillar that was expelled with faeces by a 52-year-old woman with abdominal distress.^21^ The second and third infestations of which Scott wrote were caused by larvae of moths of which only one could be identified at the genus level as *Carposina* Herrich-Schäffer (Lepidoptera: Carposinidae). The fourth case was a genito-urinary infestation by the corn earworm *Heliothis* (now *Helicoverpa*) *zea* Boddie (Lepidoptera: Noctuidae). Unfortunately, no further details are available for any of these except for the first case.

In the last century, a case was also reported in Argentina, during the first *Jornadas Entomoepidemiológicas Argentinas*, held in Buenos Aires in 1959, by the lepidopterist José Antonio Pastrana of an infant a few days old who became cyanotic when crying and had a few live *Pyralis farinalis* L. (Lepidoptera: Pyralidae) caterpillars removed from his nostrils.^22^

In December 1981, the British surgeon J. A. Smallwood described in the British Medical Journal the case of a 65-year-old woman who underwent a colostomy following an intestinal obstruction that caused constipation, pain and abdominal distension.^23^ In the days following surgery, several blackberry seeds and two dead insect larvae leaked from the surgical fistula along with the drainage fluid, a third live larva was found near the fistula. These were identified, with the help of entomologists from the London School of Hygiene and Tropical Medicine, as caterpillars belonging to the superfamily Papilionoidea.

The first and only documented case of a live caterpillar being extracted from a human wound was published in the Journal of Infectious Diseases in December 1984. It involved a 68-year-old diabetic American man who had an 8-cm necrotic ulcer on his left heel. A live leafminer caterpillar (Lepidoptera: Gracillaridae) was extracted from the ulcer.^24^ The authors were unable to determine whether the ulcer was caused by the insect or if it was a secondary infestation.

In 1987, the French infectiologist Patrice Bourée reported the case of a 19-year-old girl who had been expectorating noctuid caterpillars episodically for some years.^25^

The most recent case dates to 2023 and was reported in Italy. It concerns a 12-year-old boy with an erythematous papulopustular lesion on her chin compatible with an acneic lesion, from which a dead larva was extracted.^26^ The larva was later identified by the entomologist Moreno Dutto as a caterpillar of *Plodia interpunctella* Hubner (Lepidoptera: Pyralidae). This is also the first case where photographic evidence (of what remains) of the insect extracted from the wound is available.

## Discussion

**Figure 4.**
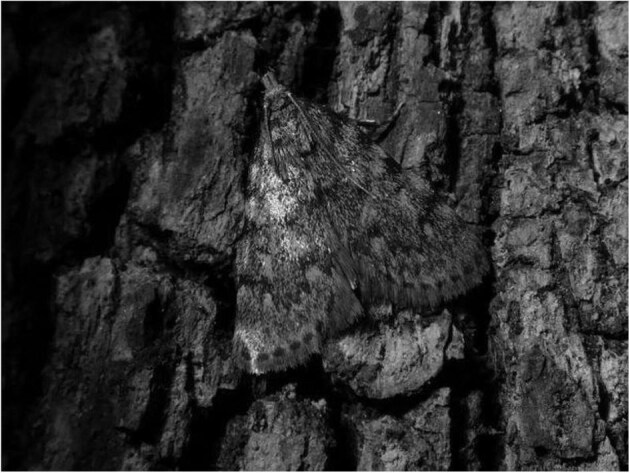
*A. pinguinalis* adult (Photo credit: Gabriele Santagati).

All the cases published between the 16th and 19th centuries and half of all cases of human infestation by lepidopteran larvae reported to date described symptoms affecting the gastrointestinal tract, such as stomach cramps, vomiting, abdominal pain, abdominal distension, constipation and diarrhoea (Table 1). Currently there is no scientific evidence to support the hypothesis that eggs of the above-mentioned or other moth and butterfly species, accidentally ingested with food, can successfully hatch inside the human digestive tract. Nor is it possible for caterpillars of any Lepidoptera species to continue their development in any way in the gut of a vertebrate, as they are not adapted to the extreme conditions of an enteroparasitic life. This was already evident to 19th-century scientists who were confronted with similar cases.^27^ Not surprisingly, all the species involved in gastrointestinal scoleciasis cases documented to date are known to be significant food pests. The reported symptomatology may have been caused by toxic substances and pathogens that contaminated the foodstuffs in which the larvae of these lepidopterans were found rather than the larvae themselves. For instance, the grease moth *A. pinguinalis*, described by Linnaeus and other of his contemporaries as a true parasite of man,^28,29^ is a species with coprophagous^30^ and necrophagous^31^ habits. Its adults (Figure 4) can be strongly attracted to the odour of butyric fermentation of fat in foods of animal origin, where they can lay eggs and the larvae (<1 mm newly hatched) can feed, grow and develop until just before pupation. Not only can the consumption of spoiled food containing *A. pinguinalis* larvae lead to serious gastrointestinal illness caused by toxic substances accumulated,^32^ but the larvae themselves can concentrate and carry enteropathogenic microorganisms.^33^

**Table 1:** Chronology and main details of the 18 known cases of human scoleciasis.

Year	Species involved	Country	Patient age/sex	Clinical manifestations	Method of larva expulsion	Reference
1610		Italy	34 y/M	Fever, headache, epistaxis and nasal congestion	Sneezing (live)	F. Angelini^12^
1675		England	9 y/M	Stomach pain and continual vomiting	Vomit (live)	M. Lister^13^
1723		France	Adult/M	Stomach cramps	Vomit	N. A. De Bois-Regard^14^
1761		France	30 y/F	Coughing fits, nausea	Paroxysmal coughing (live)	G.-L. Leclerc^15^
1783	*Pieris brassicae*? (Pieridae)	Scotland	4 y /M	Malaise, headache, bruxism, abdominal pain, comatose state	Via the anus? (live)	R. Calderwood^16^
1830	Noctua sp. (Noctuidae)	France	Adult/F	Constipation	Faeces (dead)	A. M. C. Dumeril^17^
1896	*Aglossa pinguinalis* (Pyralidae)	France	Adult/M	Nausea	Vomit	M. Poujade^18^
1896	*Aglossa pinguinalis* (Pyralidae)	France	7 y /M	Nausea, symptoms of pharyngeal foreign body	Vomit/manual removal (live)	M. Poujade^18^
1936	*Ostrinia nubilalis* (Crambidae)	Canada	58 y /F	Intense vaginal itching and irritation	Manual removal (live)	B. Church^19^
1952	Noctuidae	Canada	52 y/F	Abdominal distress	Faeces (dead)	W. W. Judd^21^
1957		USA		Gastrointestinal symptoms		H. G. Scott^20^
1959	*Pyralis farinalis* (Pyralidae)	Argentina	Infant/M	Symptoms of nasopharyngeal foreign body	Manual removal (live)	Pastrana and Duffau^22^
1961	*Carposina* (Carposinidae)	USA		Gastrointestinal symptoms		H. G. Scott^20^
1962	*Helicoverpa zea* (Noctuidae)	USA		Genito-urinary symptoms		H. G. Scott^20^
19S1	*Pieris brassicae*? (Pieridae)	England	65 y/F	Constipation, abdominal pain and distension	Via surgical wound (live)	Smallwood and Maunder^23^
19S3	Gracillaridae	USA	68 y/M	Necrotic, painless ulcer on heel (diabetic patient)	Manual removal (live)	Anderson et al.^24^
1987	Noctuidae	Algeria	19 y/F	Haemoptysis with larva expulsion	Paroxysmal coughing	Bourée et al.^25^
2023	*Plodia interpunctella* (Pyralidae)	Italy	12 y/M	Rapidly enlarging furuncle of the chin (acneic patient)	Manual removal (dead)	Cammarata et al.^26^

M: male; F: female.

In the cases described by Calderwood and Smallwood, some live *Pieris* caterpillars appear to have escaped from the anus and a colostomy wound, respectively. None of the authors can attest to being in the presence of the caterpillars at the time of emergence. However, it is theoretically possible that the indigestible cellulose of vegetables, consumed in large amounts by both patients, may have protected the larvae from the hostile stomach environment. Additionally, the caterpillar's exoskeleton, which is largely made up of chitin, a polysaccharide that is notoriously difficult for humans to digest,^34^ may have contributed to the survival of the larvae for a short period of time on their way through the digestive canal, just as can happen with undigested food or as may occur in intestinal pseudomyiasis.^35^ As in the above cases, the symptomatology in both patients does not appear to be directly related to the presence of the caterpillars in the intestine.

It is highly likely that all cases in which larvae were expectorated or extracted from the nose were caused by caterpillars ingested through contaminated food (dates, in the case described by Bourée) and either stuck in the throat or regurgitated from the stomach to the pharynx and the nasal cavity, as probably happened to the French infant described by Poujade.

There are two cases of infestation in the genitourinary tract, however, the only one with an accurate description is that reported by B. Church. The species involved is the crambid *Ostrinia nubilalis*, which is a primary pest of maize.^36^ It is unclear how the larva entered the woman's urethra and then caused the typical foreign body symptoms, but the author suggests it may have been due to accidental exposure in a context of high pest presence and/or poor personal hygiene. However, some sexual practices, such as introducing vegetables contaminated with larvae into the vagina^37^ or directly in the urethra,^38^ cannot be excluded. The other case, recorded by Pratt in 1962 and involving the wood borer *Helicoverpa zea*, lacks any details for further comment or analysis.

Two of the eighteen cases reported in this study are noteworthy as they describe the presence of caterpillars inside skin wounds, apparently exhibiting many similarities to wound myiasis caused by certain dipteran species.^39,40^ In the first case, dating back to 1984, a live leafminer larva from the Gracillaridae family was found inside a skin ulcer on the heel of a diabetic patient. Based on current knowledge of the biology of these insects, it appears very unlikely that the caterpillar actively initiated the lesion by attacking intact skin. Rather, it is plausible that somehow the larva found itself on the patient's leg at a time when there was already a skin lesion, most likely caused by the man's pre-existing medical condition (diabetes), and penetrated it, remaining trapped. It should be briefly recalled that caterpillars breathe through a series of respiratory spiracles, located in pairs laterally at the level of the abdominal segments and connected to a ramified tracheal system that allows gas exchange within the insect's tissues. Although the inside of a wound in the skin layers of a living host can be considered a hypoxic or even an anoxic environment, it has been found that caterpillars can actually survive for some time (probably several hours) in conditions of hypoxia and near-total anoxia, responding with tracheal morphological changes (sprouting) and a substantial reduction of metabolism.^41–44^ The innate ability of caterpillars to cope with extremely low levels of oxygen may be even greater in smaller species that can also surround themselves with silky hydrophobic layers, such as the gracillariid moth larva involved in this case, thereby explaining Anderson et al.’s apparently incredible finding.

In the second case, which was recorded in Italy in 2023, a larva of *P. interpunctella*, the Indian meal moth, emerged dead from a papulopustular lesion on the chin of a child suffering from acne who lived in a house in which there was a significant food storage infestation by this insect. As the authors speculate, an errant larva (which from its size had probably reached full maturity before pupation) may have accidentally found its way into a pre-existing acne lesion on the skin, being trapped inside and dying most likely by crushing or suffocation.

**Figure 5.**
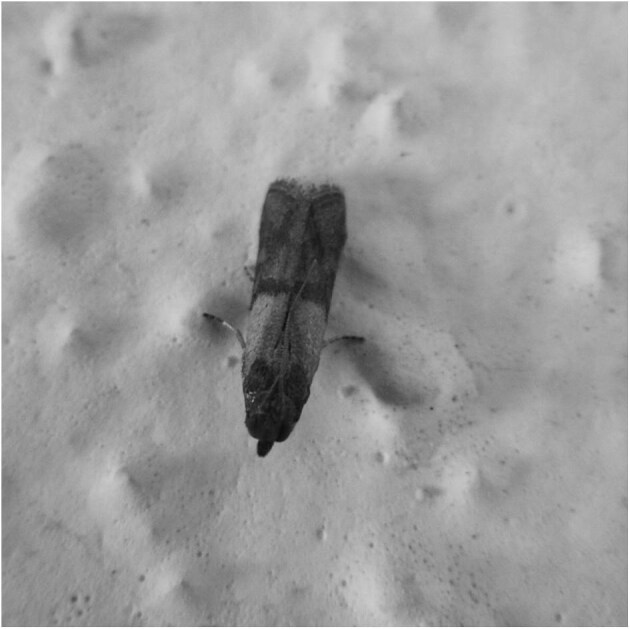
*P. interpunctella* adult.

**Figure 6.**
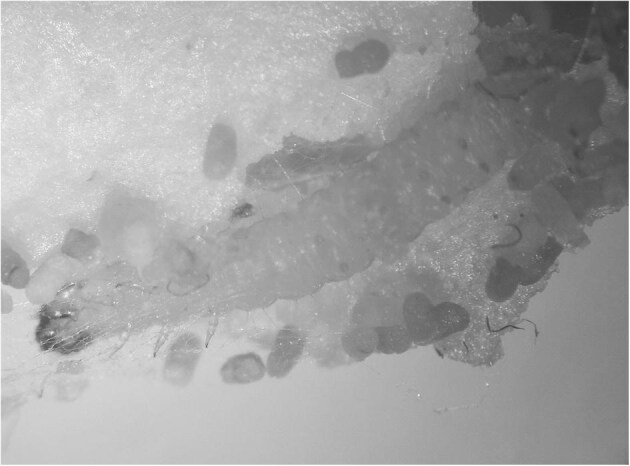
*P. interpunctella* caterpillar inside a silky layer that traps its excreta and exuviae.

Regarding *P. interpunctella*, as it was for *A. pinguinalis*, a few more words need to be said. It is a small moth (Figure 5) belonging to the family Pyralidae and is considered the most important and widespread pest of preserved and processed products on the planet.^45^ The primary reason for its widespread success is its remarkable adaptability to human environments, as evidenced by its recent discovery in an Antarctic base,^46^ and its ability to feed on a wide variety of food sources.^47,48^ However, the invasive capacity of the Indian meal moth is perhaps its most impressive feature. The larvae are able to pass through even the smallest holes in food packaging and the advanced stages are capable of actively penetrating intact packages using their powerful jaws.^49,50^ These larvae have a distinctive feature of continuously producing a silky web that traps their excreta and exuviae (Figure 6). This web contributes to damaging the product they are feeding on by creating a microenvironment that promotes the growth of moulds and other organisms that are known to be hazardous to human health.^51^ The larvae move away from the food source before pupating, often travelling a considerable distance. This behaviour, especially in heavily infested environments, can lead to them getting on people's clothes and being found eventually on their skin.

In 2001, the Journal of Medical Entomology published an article about the cases of infestation by *P. interpunctella* larvae in a wild-caught Indian ring-necked parakeet and two domestic cats.^52^ In the parakeet, the insect had found a way in and then migrated to the skull, where it was extracted during the autopsy of the bird, which had died of a concurrent viral infection. At the time of extraction, the larva was still alive and covered by its silky web. The necropsy did not reveal any macroscopic signs of tissue erosion, as is the case with some helminth infestations (visceral larva migrans) or in some cases of nasopharyngeal myiasis in sheep.^53^ It is possible that the larva, having entered the host through the nostrils during a meal of contaminated food, then migrated through the narrow passages to reach the inside of the skull. In the two domestic cats, the larvae were found and extracted intradermally, in the context of diffuse scratching lesions, suggesting, as mentioned above, that the insects had entered pre-existing wounds.

From a parasitological point of view, it is important to note that the spurious passage of non-parasitic organisms through the human digestive tract—as described in all cases of accidental caterpillar ingestion—is a phenomenon already described in the medical literature.^54,55^ Since its first use in 1870 by Lacordaire, the correct term to define these organisms is ‘pseudoparasites’, from the ancient Greek ‘ψευδο’ (pseudo) = ‘false’, false parasites.^56^ Every ‘gastrointestinal’ scoleciasis is therefore a pseudoparasitosis, analogous to ‘intestinal myiases’, which are, in fact, pseudomyiases.^35^

Regarding those exceptional and anecdotal cases where caterpillars of moth species considered to be primary food pests have been found in body cavities or wounds of humans (and animals), these appear to be the result of errant larvae in search of a pupation site incidentally finding a way in and then simply becoming trapped, with no clear evidence of harmful effects in any of the reported events. The availability of oxygen in the intratissue environment and the oxygen remaining in the insect's trachea are most likely to be the main limiting factors for larval survival in these hypoxic/anoxic conditions. To date, there is no convincing evidence that a caterpillar can feed, actively invade tissues or further develop inside a living vertebrate host, all essential conditions for defining a parasite and the disease it causes a true ‘parasitosis’. In fact, even the most controversial cases of ‘wound’ and ‘urogenital scoleciasis’ should be considered only pseudoparasitoses. That is why this condition has never found its place among human parasitoses.^57,58^

## Conclusions

Describing the phenomenon of scoleciasis is difficult because of its rarity: only 18 cases have been reported in humans in the last 4 centuries. Given the widespread occurrence of some of the species involved, this paucity of data alone may be enough to cast doubt on the authenticity of the condition as a ‘parasitic infestation’ as originally interpreted by Kirby and Spence in 1815.

In the scientific literature, the terminology on the subject is often confused and misused, and although the word *scoleciasis* originated contemporaneously with that of *myiasis* almost 2 centuries ago, the latter has evolved and reshaped over time, being enriched with details and classifications, leaving the first behind and basically unchanged.

In some of the publications reviewed in this study, the juxtaposition of the real pathogenic role of certain fly larvae as agents of myiasis with the unproven assumption of a similar potential in the caterpillars of certain moth species may have contributed to the erroneous belief that scoleciasis, like myiasis, is a true parasitic infestation. Instead, it should be emphasized that the two phenomena are very different and seem to have no substantial overlaps.

In the light of what has been said so far, the choice made by the modern parasitology community and matured over the last century not to include Lepidoptera as human parasites seems to remain correct.

## Data Availability

No new data were generated or analysed in support of this research.
